# Anxiolytic effects of chewing gum during preoperative fasting and patient-centered outcome in female patients undergoing elective gynecologic surgery: randomized controlled study

**DOI:** 10.1038/s41598-022-07942-6

**Published:** 2022-03-09

**Authors:** Yu Jeong Bang, Jong-Hwan Lee, Chung Su Kim, Yoo-Young Lee, Jeong-Jin Min

**Affiliations:** 1 Department of Anesthesiology and Pain Medicine, Samsung Medical Center, Sungkyunkwan University School of Medicine, 81 Irwon-ro, Gangnam-gu, Seoul, 06351 Korea; 2 Division of Gynecologic Oncology, Department of Obstetrics and Gynecology, Samsung Medical Center, Sungkyunkwan University School of Medicine, Seoul, Korea

**Keywords:** Quality of life, Anxiety

## Abstract

Although previous studies reported that chewing gum during the preoperative fasting has the benefits of alleviating anxiety and dry mouth, preoperative chewing gum has yet to be accepted as a standard practice due to conventional anesthetic custom. Our study aimed to prospectively evaluate the effects of gum chewing on preoperative anxiety and patient’s discomfort in female patients undergoing gynecologic surgery. Ninety-four patients were enrolled and randomized either into conventional fasting group (control group) or chewing gum with fasting group (gum group). The control group was instructed to fast from 3 p.m. on the day before surgery. The gum group performed preoperative fasting in the same manner, but was encouraged to chew gum freely during the fasting period. The primary endpoint was the degree of preoperative anxiety. For the evaluation of preoperative anxiety, Amsterdam preoperative anxiety and information scale (APAIS) was used. Preoperative gastric fluid volume and acidity were also measured as the secondary outcomes. Preoperative anxiety using APAIS was significantly lower in the gum group compared to the control group (control group vs. gum group: 20.9 vs. 17.8, *p* = 0.009). However, there was no significant difference in the gastric fluid analysis between the groups. In the female patients for elective gynecologic surgery, chewing gum during the preoperative fasting period helped to alleviate preoperative anxiety without additional increase of pulmonary aspiration risks.

*Trial registration*: KCT0004422 (05/11/2019, https://cris.nih.go.kr; registration number).

## Introduction

*‘Nil per OS’* (NPO) is a prerequisite for elective surgery to minimize the risk of pulmonary aspiration and associated complications^1^. Fasting before general anesthesia reduces the volume and acidity of gastric contents and ensure reducing the risk gastric regurgitation and severity of pulmonary aspirations^2,3^. Currently, most authoritative guidelines suggest relaxed fasting rule to prohibit solid food for 6 h and clear liquids for 2 h before elective surgery^1,2,4,5^. However, depending on the progress of surgical procedures in the operating room, the NPO period tends to be longer than expected, and this prolonged fasting may increase preoperative discomfort or anxiety^6^.

Chewing gum has the effect of stress reduction, amelioration of dry mouth, and promote gastrointestinal motility^7–9^. Because gum has been treated as solid food by anesthesiologists, many institutional NPO guidelines forbid chewing gum during preoperative fasting, and some institutions recommend it only in the postoperative period to improve bowel recovery^10–14^. However, in several previous studies, preoperative gum-chewing did not increase the gastric content volumes and the risk of pulmonary aspiration^15–17^. Based on this, some opinions have recently emerged to allow or recommend chewing gum during the preoperative fasting period focusing on the potential benefits of alleviating anxiety and dry mouth^16,18^.

Preoperative anxiety is linked to increase in anesthetic requirements and consumption of postoperative analgesics. In addition, increased level of preoperative anxiety has been proved to negatively affect both psychological and somatic aspect in postoperative care^19^. Therefore, increased preoperative anxiety may adversely influence patient recovery and satisfaction^19–22^. Moreover, female patients, especially those undergoing gynecological surgery, are known to have increased preoperative anxiety compared to male patients or female patients undergoing other minor or general surgery^23–26^. Thus, it is necessary to address the preoperative anxiety in this patient population. However, most studies of preoperative chewing gum have focused on its effects on gastric volume and acidity, and clinical data regarding the patient-centered benefits of alleviating preoperative anxiety and discomfort are still insufficient.

In this randomized clinical study, we aimed to evaluate the effects of chewing gum on preoperative anxiety and discomfort in female patients undergoing elective gynecologic surgery.

## Results

**Figure 1:**
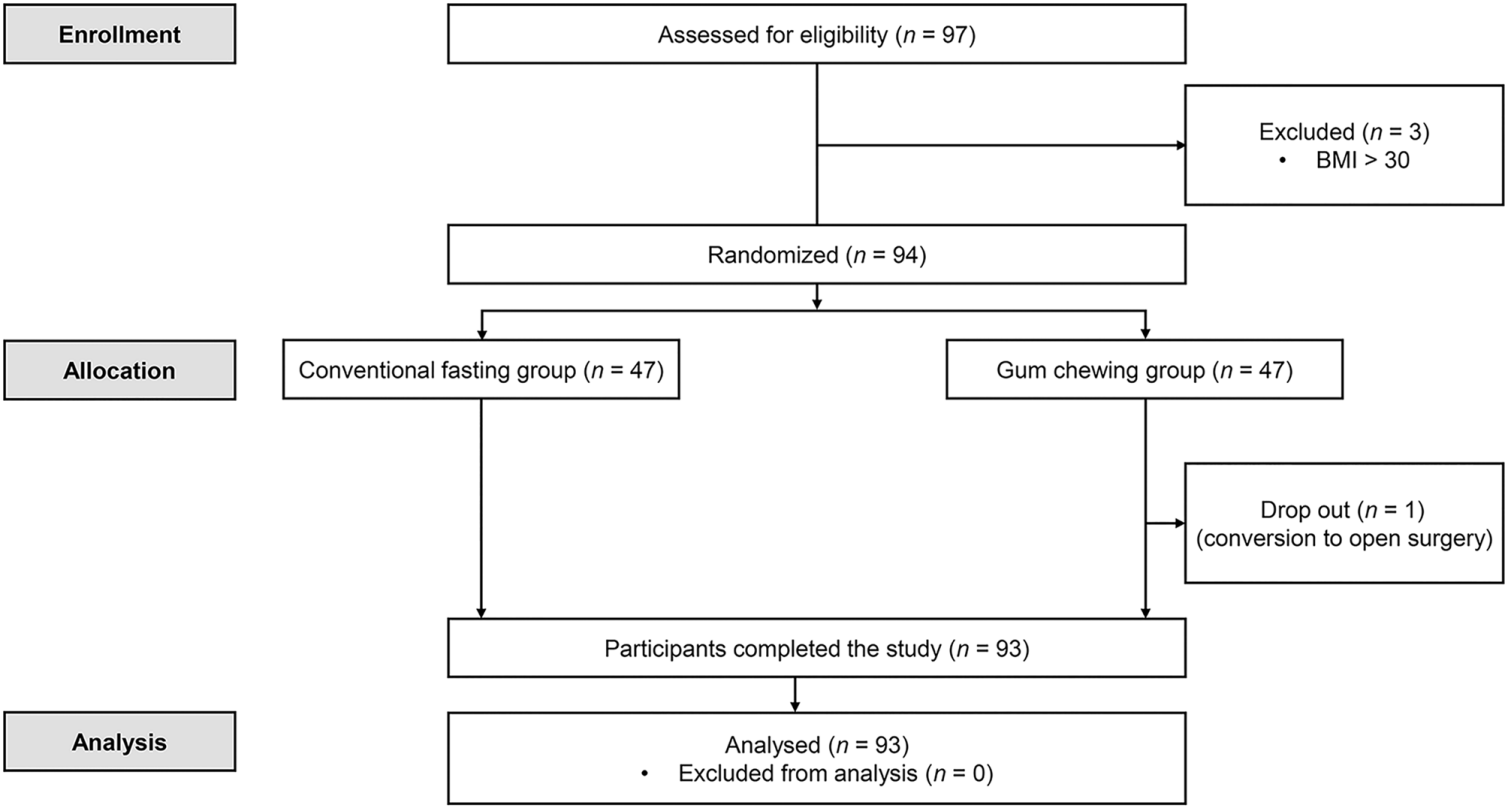
CONSORT flow diagram of patients included in the study. The conventional fasting group comprised patients who followed conventional preoperative fasting guidelines. The gum chewing group comprised patients who allowed to chew gum freely during the preoperative fasting period. *CONSORT* consolidated standards of reporting trials.

A total of 97 patients were screened for eligibility. After excluding 3 patients (BMI > 30), 94 patients were randomized either into the control group or gum group. One patient in the gum group was excluded due to unexpected conversion of surgical approach to open laparotomy. Consequently, 93 patients (47 patients in the control group and 46 patients in the gum group) completed the study and were analyzed (Fig. 1). The demographic and perioperative data are summarized in Table 1. No differences were observed between groups in terms of fasting time or intraoperative data. The median value of chewing time in the gum group was 110 min [IQR 57–180].

**Table 1:** Patient characteristics and perioperative data.

Parameter	Control group (*N* = 47)	Gum group (*N* = 46)
Age (years), mean (SD)	42.9 (10.2)	43.8 (10.9)
Height (cm), mean (SD)	159.2 (5.0)	160.3 (6.0)
Weight (kg), mean (SD)	59.5 (8.4)	57.8 (6.9)
Body mass index (kg m^−2^), median [IQR]	22.5 [21.1–25.9]	22.4 [21.0–24.0]
ASA physical status (I; II), *n*/total *N* (%)	27/47 (57%); 20/47 (43%)	27/46 (59%); 19/46 (41%)
**Occupation, *n*/total *N* (%)**		
Housewife	21/41 (51%)	21/42 (50%)
Clerical worker	10/41 (24%)	9/42 (21%)
Service worker	4/41 (10%)	2/42 (5%)
Professionals	3/41 (7%)	6/42 (14%)
Student	0/41(0.0%)	2/42 (5%)
Retired or unemployed	3/41 (7%)	2/42 (5%)
**Marital status, *n*/total *N* (%)**		
Married	36/47 (77%)	30/46 (65%)
Unmarried, divorced, or bereaved	11/47 (23%)	16/46 (35%)
Malignancy, *n*/total *N* (%)	17/47 (36%)	19/46 (41%)
**History of previous surgery, *n*/total *N* (%)**		
0	13/47 (28%)	12/46 (26%)
1–2	31/47 (66%)	28/46 (61%)
≥ 3	3/47 (6%)	6/46 (13%)
**History of previous anesthesia, *n*/total *N* (%)**		
General anesthesia	22/47 (47%)	22/46 (48%)
Regional anesthesia	10/47 (21%)	9/46 (20%)
MAC	2/47 (4%)	3/46 (7%)
Anxiety about upcoming surgery and anesthesia procedures, median [IQR]	2 [0–5]	2 [0–5]
Fasting time (h), median [IQR]	19.7 [18.0–21.6]	21.7 [18.3–24.1]
**Extents of surgery, *n*/total *N* (%)**		
Ovarian cystectomy	4/47 (9%)	9/46 (20%)
Salpingo-oophorectomy	8/47 (17%)	9/46 (20%)
Myomectomy	8/47 (17%)	7/46 (15%)
Hysterectomy	24/47 (51%)	17/46 (37%)
Miscellaneous	3/47 (6%)	4/46 (9%)
**Surgical access, *n*/total *N* (%)**		
Single port laparoscopic surgery	7/47 (15%)	6/46 (13%)
Dual port laparoscopic surgery	14/47 (30%)	23/46 (50%)
Conventional laparoscopic surgery	15/47 (32%)	16/46 (35%)
Robot assisted laparoscopic surgery	11/47 (23%)	1/46 (2%)
Anesthetic time (min), mean (SD)	144.0 (51.1)	133.4 (38.7)
Crystalloid (mL), mean (SD)	778.7 (303.9)	685.9 (226.2)
Estimated blood loss (mL), median [IQR]	50 [50–100]	100 [50–150]
Urine output (mL), median [IQR]	100 [0–120]	50 [0–120]
Opioid (MED), median [IQR]	6.01 [3.35–9.34]	6.64 [3.33–10.02]

Anxiety about upcoming surgery and anesthesia procedures was rated using a numeric rating scale (NRS), 0–10.

*ASA* American Society of Anesthesiologists, *IQR* interquartile range, *MAC* monitored anesthesia care, *MED* morphine equivalent dose, *SD* standard deviation. Denominators that do not equal the sample sizes are due to missing data.

**Figure 2:**
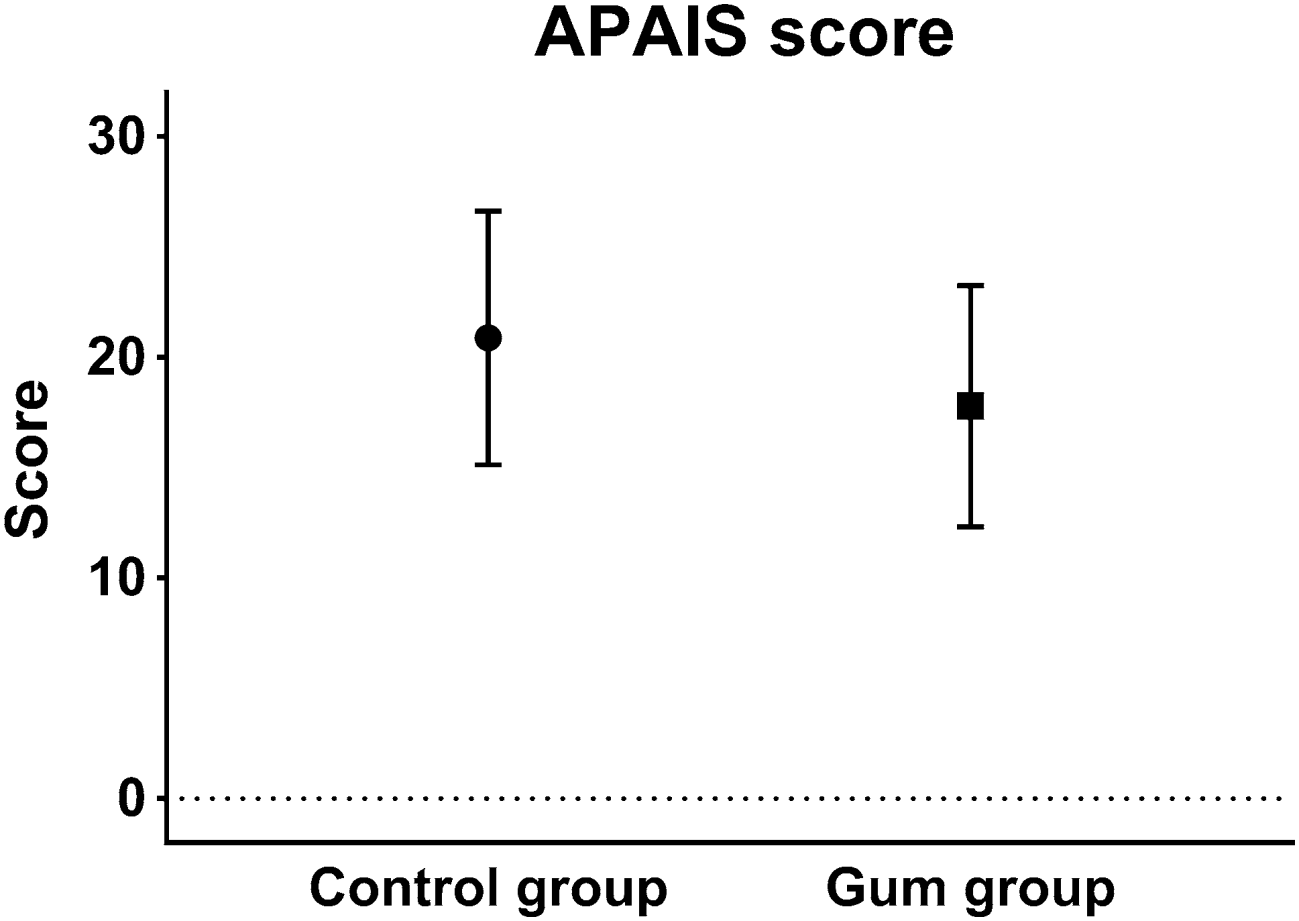
Comparison of patient anxiety scores using APAIS (the Amsterdam Preoperative anxiety and information Scale) before surgery. Data were analyzed using student’s *t* test.

The degree of anxiety assessed using APAIS is shown in Fig. 2. The mean value (SD) of total APAIS was significantly lower in the gum group compared to the control group (control group vs. gum group: 20.9 [5.7] vs. 17.8 [5.5], *p* = 0.009). However, patient discomfort related to preoperative fasting such as hunger, thirst, dry mouth, fatigue, headache, and nausea did not differ between groups (Table 2). There was no significant correlation between chewing time and preoperative discomfort and anxiety, but only weak inverse correlation with headache (Spearman correlation coefficient = − 0.034, *p* = 0.022).

**Table 2:** Preoperative patient discomfort and gastric contents analysis.

Parameter	Control group (*N* = 47)	Gum group (*N* = 46)	Difference in means or medians (95% CI)	*p* value
APAIS—total, mean (SD)	20.9 (5.7)	17.8 (5.5)	3.1 (0.8 to 5.4)	0.009^a^
APAIS—anxiety, mean (SD)	14.1 (4.1)	12.3 (3.8)		
APAIS—information desire, median (IQR)	7 [6–8]	6 [4–7]		
**Preoperative discomfort**				
Hunger, median [IQR]	2 [0–5]	3 [0–5]	− 1 (− 3.0 to 1.0)	0.65^b^
Thirst, median [IQR]	4 [0–5]	3 [1–5]	1 (− 0.7 to 2.7)	0.85^b^
Dry mouth, median [IQR]	4 [1–5]	3 [1–5]	1 (− 0.7 to 2.7)	0.43^b^
Fatigue, median [IQR]	3 [0–6]	3.5 [1–5]	0 (− 2.2 to 2.2)	0.86^b^
Headache, median [IQR]	0 [0–7]	1 [0–4]	− 1 (− 3.0 to 1.0)	0.80^b^
Nausea, median [IQR]	0 [0–2]	0 [0–1]	0 (− 0.8 to 0.8)	0.13^b^
**Oral secretion**			Absolute risk difference (95% CI)	0.94^c^
None	2	2	− 0.1 (− -8.3 to 8.2)	
Mild	34	32	− 1.5 (− 20.0 to 16.9)	
Moderate	10	10	3.8 (− 12.8 to 20.5)	
Severe	1	2	− 2.2 (− 9.4 to 5.0 )	
Gastric pH, median [IQR]*	1.34 [0.42–2.78]	1.45 [0.55–2.2]	− 0.10 (− 1.10 to 0.90)	0.95^b^
Estimated gastric fluid volume (ml kg^−1^), median [IQR]^†^	0.14 [0–0.58]	0.24 [0–0.62]	− 0.09 (− 0.35 to 0.17)	0.70^b^

Patients’ discomfort related to preoperative fasting was rated using a numeric rating scale (NRS), 0–10.

*APAIS* the Amsterdam preoperative anxiety and information scale.

*Data were available from 37 patients in the control group and 36 patients in the gum group. The gastric fluid was collected via ST probe by gravity drainage without suction, as a minimally invasive method.

^†^Data were available from 45 patients in the control group. The unavailable two cases were as follows: one case with the antrum was obscured by the colon and the other being difficult to †measure due to peristalsis.

^a^Student’s *t* test.

^b^Wilcoxon rank sum test.

^c^Fisher’s exact test.

Regarding gastric fluid volume and acidity, there were no significant differences between the two groups. The amounts of oral secretion graded during tracheal intubation also did not differ between groups.

Postoperative bowel recovery, bowel complication during the in-hospital period, and LOS did not show significant difference between groups (Table 3). However, QoR-15 score, representing the subjective recovery, was significantly higher in the gum group.

**Table 3:** Postoperative outcomes of bowel function recovery.

Parameter	Control group (*N* = 47)	Gum group (*N* = 46)	Difference in means or medians (95% CI)	*p* value
Time to flatus (h), median [IQR]*	26.4 [15.2–39.2]	20.6 [16.8–38.9]	5.4 (− 6.2 to 17.1)	0.53^a^
**Total bowel complication,** ***n*/total** ***N* (%)**	32/47 (68%)	23/46 (50%)	Absolute risk difference (95% CI) 18.1 (− 1.6 to 37.7)	0.08^b^
Nausea	26	20	11.8 (− 8.3 to 32.0)	0.25^b^
Vomiting	3	6	− 6.7 (− 18.6 to 5.3)	0.32^c^
Postprandial pain	8	3	10.5 (− 2.4 to 23.4)	0.12^b^
Abdominal distension	0	2	− 4.4 (− 10.2 to 1.6)	0.24^c^
Clavien Dindo classification				> 0.999^c^
Class 1	31	22	1.2 (− 9.1 to 11.5)	
Class 2	1	1	− 1.2 (− 11.5 to 9.1)	
Rescue antiemetics, median (IQR)	0 [0–1]	0 [0–1]	0.0 (− 0.5 to 0.5)	0.32^a^
Discharge delay, *n*/total *N* (%)	4/47 (9%)	2/46 (4%)	4.2 (− 5.8 to 14.1)	0.68^c^
QoR—15 score, mean (SD)	96.8 (27.7) *n* = 37	116.0 (21.0) *n* = 36	− 19.2 (− 30.7 to − 7.6)	0.001^a^
Hospital stay (day), median [IQR]	2 [2, 3]	2 [1–3]	0.0 (− 0.8 to 0.8)	0.22^a^

*Data were available from 34 patients in the control group and 30 patients in the gum group. Patients could be discharged even before the postoperative gas out was confirmed if there were no gastrointestinal symptom, according to our gynecological policy.

^a^Wilcoxon rank sum test.

^b^Chi-squared test.

^c^Fisher’s exact test.

## Discussion

In this randomized controlled study, chewing gum during preoperative fasting alleviated preoperative anxiety and promoted patient-reported quality of postoperative recovery, without increasing the risk of pulmonary aspiration in female patients undergoing gynecological surgery.

In the current study, patients who chewed gum showed significantly lower anxiety levels in the preoperative holding area, consistent with previous researches. The anxiolytic effect of chewing gum may be attributed mainly to the act of chewing. Mastication and sham feeding in stressful situations have been shown to decrease levels of plasma cortisol and stress-related substances including neurotrophic factors, through the modulation of the hypothalamic–pituitary–adrenal axis and autonomic nervous system, especially in humans with acute stress^27–30^. In particular, chewing gum increases attentiveness, improves mood, and reduces stress and anxiety^28,31–33^. It is thought that chewing gum during the preoperative period may relieve emotional tension or stress, resulting in lower preoperative anxiety levels. The preoperative NPO time in this study was longer than necessary due to the policy of our gynecology department. Patients might have felt liberated by being allowed to chew gum during the prolonged NPO.

Preoperative anxiety is influenced by multiple factors such as patient personality, past experiences, or education level^23,25,34^. Therefore, an individualized approach is needed to alleviate anxiety, which requires additional medical personnel or costs. However, preoperative gum chewing can be used easily as a strategy to relieve anxiety for almost all patients, without the consumption of expensive medical resources. In addition, the reduced immediate pre-operative anxiety appears to be associated with a higher patient-reported quality of recovery score evaluated by QoR—15, which may be interpreted to indicate the patient's sense of well-being in the perioperative period.

The average level of preoperative anxiety in our study was higher than previously reported by other investigations. The mean value of total APAIS in previous studies varies from 8.31 to 14.50^23,35–37^. The participants in the present study showed mean APAIS value of 19.3 (5.8) in this study. We consider the reasons for inconsistency between our findings as follows. In previous studies, women, gynecological surgery, and cancer surgery were cited as important factors for preoperative anxiety^23,36^. All participants of our study were female patients undergoing gynecological surgery, and 41% of them were already diagnosed as cancer or BRCA gene (+). Second, according to previous studies, anxiety increased as the operation approached^38,39^. In our study, anxiety was measured immediately before entering the operating room, whereas previous study investigated preoperative anxiety at outpatient clinics for pre-anesthetic evaluation a few days prior to surgery. Jiwanmall et al.^38^ investigated the preoperative anxiety in patients on the day of surgery and reported that mean value of APAIS-A was 13.8 (3.54) and mean value of APAIS-ID was 7.17 (1.72), which was similar result with this study. In addition, we consider that the excessively long fasting time also affected increasing preoperative anxiety^6^. Before surgery, patients are often in a state of acute stress and are thus prone to anxiety. Leptin, the satiety hormone is decreased on exposure to stressful environments^40^, which makes patients hungrier during preoperative fasting. Since fasting is somewhat contrary to human nature, preoperative fasting would be an even more unpleasant experience for patients undergoing surgery and may increase preoperative discomfort and anxiety^6^.

The major concerns related to chewing gum during preoperative fasting is possibly increased risk of pulmonary aspiration by increased gastric fluid volume and acidity^16,17,41–44^. In the present study, factors that could affect pulmonary aspiration risk such as saliva secretion, gastric fluid volume, and gastric fluid acidity remained unchanged after gum chewing. Gastric volume of up to 1.5 mL kg^−1^ is reported to be within the normal range in fasted adults^45,46^, and 97% of subjects in this study had a completely empty stomach (≤ 1.5 ml kg^−1^). The mean value of gastric volume was 0.4 ml kg^−1^ in both groups. In terms of gastric acidity, gastric fluid pH was not different between the groups.

An important precaution when patients are allowed to chew gum during the preoperative NPO period is to ensure that the patient removes gum from the mouth before induction of anesthesia. In our protocol, participants were instructed to dispose of gum before departure from the ward on the day of operation. There were no adverse events caused by chewing gum such as airway obstruction, but one patient chewed gum in the preoperative holding area. If patients chew gum during pre-anesthetic fasting, there should be a step to ensure that the gum has been removed before entering the operating room.

Our study has clinical implications in the following aspects. The data collection was prospective in nature and conducted by a clinician not involved in postoperative patient care. While previous studies have focused on the safety of chewing gum in preoperative fasting, the current study evaluated the effects of chewing gum in preoperative fasting on patient anxiety, discomfort, satisfaction, and postoperative recovery from various angles, confirming safety as well. In particular, it has the advantage of presenting the variables of postoperative recovery comprehensively, from patient subjective reports to objective indicators. Our study is meaningful in that the quality of recovery was evaluated both from the perspective of the patients and the medical staff.

Despite these strengths, this study has several limitations. The main limitation is the lack of blinding. The participants could not be blinded to their group allocation due to the nature of the study. The second limitation is that there was no active control group in this study. Because different expectations of the participant according to the group allocation (control group vs. intervention group) may affect the outcome measurement, it would be more appropriate to set up an active control group to determine whether chewing gum has a distinguishing effect compared to the active comparator (such as anxiolytic premedication or other non-pharmacologic anxiolytic interventions). Third, we did not measure the baseline APAIS score, which could confound our findings. The APAIS is a six-item questionnaire with two subdomains (APAIS-A: anxiety about anesthesia and surgical procedures; and APAIS-ID: desire for information). We believed that there could be variation in the amount of information provided about the surgery to patients at the time of the first clinical visit. Therefore, to simplify the process, we used the modified NRS form to assess “anxiety about upcoming surgery and anesthesia procedures”, which could be used instead of the four items of the APAIS-A sub-scale. NRS and visual analogue scales have also been used as reliable measures of preoperative anxiety and the severity of specific fears^23,47,48^. Although, it cannot replace the baseline APAIS score, we believe that the NRS adequately reflects patient anxiety before surgery, which was comparable between our groups. Forth, participants were limited to female adults with ASA physical status I–II scheduled gynecologic laparoscopic surgery. It is difficult to generalize the results of the current study to pediatric, elderly, and specific disease cohorts. Although we verified that chewing gum has positive effects on preoperative anxiety and promotes postoperative recovery without further risk, future research is needed to investigate whether chewing gum is beneficial, especially for patients with increased pulmonary aspiration risk. Last, in this study protocol, chewing gum was encouraged freely only prior to surgery without standardized instruction, so it is difficult to determine whether gum had the effect of promoting bowel recovery, which has been reported as an advantage of chewing gum after cesarean section, gynecologic surgery, and colorectal surgery^49–51^. There is a possibility that the potential benefits of chewing gum in preoperative fasting was attenuated because the preoperative NPO time was longer than necessary due to the internal policy of our hospital's gynecology department. Future research is needed on the integrative benefits of pre- and postoperative gum chewing in the perioperative period. Nevertheless, chewing gum in preoperative fasting had positive effects in reducing anxiety and promoting recovery in this study.

## Conclusion

This randomized controlled trial in female patients undergoing elective gynecologic surgery demonstrated that chewing gum during the preoperative fasting period helped to alleviate preoperative anxiety without affecting gastric volume or pH values. Further studies are needed examining the effects of preoperative chewing gum on other patient populations in groups of different aged or in different perioperative conditions.

## Methods

### Study design

This study is a single-center prospective randomized controlled study. The study was conducted in accordance with the principles of the Declaration of Helsinki and the International Conference on Harmonisation of Good Clinical Practice guidelines. The study protocol was approved by the institutional review board of Samsung Medical Center (IRB number: SMC 2019-05-168-001) and registered at CRIS (https://cris.nih.go.kr; registration number KCT0004422; 05/11/2019). We enrolled female patients scheduled for elective gynecologic laparoscopic surgery between August 2019 and June 2020. Patients were included if they were 19–70 years of age with American Society of Anesthesiologists physical status I–II. Patients at increased risk for pulmonary aspiration were excluded. Exclusion criteria were as follows: emergency surgery, body mass index (BMI) > 30, previous esophageal or gastric surgery, gastroesophageal reflux, gastrointestinal disorders (including gastritis, hiatal hernia, diabetic gastroparesis), ileus and current medication affect gastrointestinal motility^52–54^. Eligible patients were randomly assigned to either conventional fasting group (control group) or chewing gum with fasting group (gum group) at a ratio of 1:1, according to a randomization list generated by random permuted block design with a block size of two.

### Intervention and anesthesia

The internal policy of gynecologic department of our hospital had stipulated the guidelines about preoperative fasting and bowel preparation as follows: All patients scheduled for gynecologic surgery were instructed to ban solid food from 3 p.m. the day before surgery and clear liquids from midnight before surgery according to protocol of the department of gynecology. In addition, 170 mL of Picosolution® (Pharmbio Korea Inc: Seoul, South Korea) was administered orally for bowel preparation at 5 p.m. on the day before surgery. Our patients were hospitalized around 5 p.m. the day before surgery and invited to participate in this study. After obtaining informed consent, the patients were asked to rate their preoperative anxiety, as a baseline measure, on a self-assessment NRS (numeric rating scale); 0 = calm/no anxiety to 10 = extreme anxiety about the upcoming surgery and anesthesia procedures (Supplementary Figure 1). Patients in the control group were requested to follow the fasting guidelines without further treatment. Patients in the gum group were allowed to chew gums during the fasting period, following the fasting protocols described above. We distributed 12 pieces of sugarless xylitol gum (Xylichew: Hayden, Idaho, USA) to each participant. We asked participants to chew gum during preoperative fasting period and to stop chewing gum from departure time for the operating room on the day of surgery. Participants were basically instructed to chew gum freely. It was recommended to chew gum at least one piece of gum more than 10 min per hour, except for sleep time. All participants in gum chewing group were asked to log their chewing time. On the day of surgery, all patients were instructed to remove any gum from their mouths immediately prior to departure for the operating room, and complete questionnaire about the degree of discomfort associated with fasting and anxiety in the preoperative holding area with the help of attending residents or nurses in anesthesia team who were not aware of group allocation. In the operating theater, just before general anesthesia was induced, US assessment of gastric fluid volume was performed in both groups. All ultrasound (US) exams for estimating gastric volume were performed by independent blinded investigator (YJ, Bang), who was instructed and trained by an experienced radiologist.

Then, a standardized anesthesia protocol was used for all patients. The standard ASA monitoring including noninvasive blood pressure, EKG, pulse oximetry, and bispectral index (BIS) monitoring was applied. After denitrogenation with 80% oxygen, general anesthesia was induced with propofol and remifentanil using target-controlled infusion (Orchestra® Base Primea; Fresenius Kabi, Brezins, France), and intravenous rocuronium 0.8 mgkg^-1^. Another investigator who was not aware of group allocation performed intubations and evaluated the degree of oral secretion during intubation. After tracheal intubation via video stylet, the ventilator was set with a tidal volume of 8 mLkg^-1^of ideal body weight and FiO_2_ 40%. The respiratory rate and I:E ratio were adjusted to maintain inspiratory peak pressure less than 30 cmH_2_O and normocapnia. During the whole surgery, propofol and remifentanil effect site concentrations were adjusted to achieve BIS values of 40–50 and to maintain mean blood pressure and heart rate within 20% of pre-induction values. At the end of surgery, the neuromuscular block was reversed with pyridostigmine (250 mcgkg^−1^) and glycopyrrolate (10 mcgkg^−1^). After confirming that spontaneous breathing was sufficient and consciousness had returned, tracheal extubation was performed.

### Postoperative management

Postoperative recovery and bowel complication during the in-hospital period were evaluated and documented by independent gynecologists. The postoperative analgesia was standardized for all patients. If patients presented with breakthrough pain (NRS ≥ 4/10), IV ibuprofen 400 mg was administered. If this proved ineffective after 30 min IV pethidine 50 mg was administered. Postoperative nausea and vomiting were treated with intravenous metoclopramide 10 mg and ramosetron 0.3 mg. All patients resumed diet and ambulation as soon as possible after full recovery from anesthesia unless gastrointestinal symptoms were noted. Hospital discharge was determined by the surgery team.

### Data collection and outcomes

**Figure 3:**
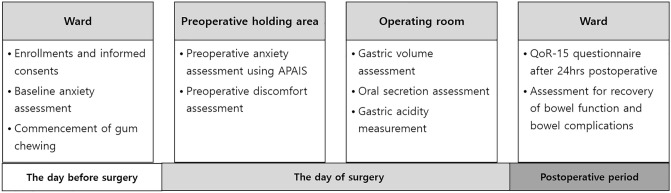
Experimental protocol during study period.

Primary outcome was the preoperative anxiety using APAIS immediately before entering OR. We investigated preoperative anxiety using the Korean version of Amsterdam Preoperative Anxiety and Information Scale (APAIS)^55,56^. APAIS is a useful tool to evaluate preoperative stress and anxiety, which consists of an anxiety scale and a need for information scale. The scores on the anxiety of APAIS range from 4 (not anxious) to 20 (extremely anxious). We also compared the severity of symptoms related to preoperative fasting between groups. The parameters used to assess discomfort were as follows: hunger, thirst, dry mouth, fatigue, headache, nausea using an 11 point NRS; 0 = no suffer to 10 = worst suffer imaginable^57^. For gum group only, the satisfaction with chewing gum during the preoperative fasting period was investigated. Secondary outcomes included the amount of oral secretion observed during tracheal intubation, estimated gastric volume just prior to induction of anesthesia, gastric fluid acidity, recovery of bowel function, composite of postoperative bowel complication, QoR-15 score (Quality of Recovery—15), and the length of hospital stay (LOS). The amount of oral secretion was graded depending on the degree of interference with intubation as follows; none (no saliva, thick tongue or mucosa causing the endotracheal tube stuck), mild (some saliva with good visual field), moderate (much saliva with limited field), severe (much saliva with blocked field, need for suction). For evaluation of recovery of bowel function, we collected data about time to flatus. We collected the data about postoperative bowel complication such as nausea, vomiting, abdominal pain, and abdominal distension. The severity of postoperative bowel complication was classified according to Clavien Dindo classification^58^. On the day after surgery, patients completed Korean version of QoR-15 questionnaire, which provides an extensive effective evaluation of quality of recovery after surgery and anesthesia^59^. Each time- points of outcome measurement was shown in detail with flowchart (Fig. 3).

### Acidity of gastric fluid analysis

ST probe 12Fr G type (SST12; S&S med Inc; Anyang, South Korea) was inserted trans-orally to monitor the core body temperature. ST probe is a multi-orificed silicone tube with suction hole for gastric decompression. After induction of anesthesia, we measured the length from the mouth to the mandible angle, then from the mandible angle to the midpoint between xiphoid and the umbilicus. Then ST probe was advanced through oropharyngeal airway to the previously identified length and the end tip of the ST probe was placed in the stomach. Patients were placed in extreme head down position by surgeon’s request so that gastric fluid could be drained via suction hole, naturally. If gastric fluid does not flow out by the end of surgery, the patient is tilted to the left to promote gastric fluid drainage. The acidity of the gastric fluid was analyzed with pH meter (PH60F Flat PH tester; Apera, Columbus, Ohio, USA) by blinded investigator.

### Gastric ultrasonography

The aforementioned-investigator (YJ, Bang) performed US assessment using a portable US unit (Sonosite M- TURBO, Fujifilm Sonosite, Bothell, WA, USA) with a 2–5 Hz convex probe. All patients underwent US exams of the epigastrium in the right lateral position. The gastric antrum was identified between the left lobe of the liver and the pancreas, at the level of the aorta, or the inferior vena cava. The cross-sectional area (CSA) was measured from serosa to serosa using the free tracing tool of the ultrasound machine. If the antrum had a perfect elliptical shape, CSA was calculated using the following formula: CSA (cm^2^) = (anteroposterior diameter [cm] × craniocaudal diameter [cm] × π)/4. Estimated gastric fluid volume (EGFV) was calculated using the following formula^54^: EGFV (mL) = 27.0 + 14.6 × Right lateral CSA – 1.28 × age.

### Sample size calculation and statistical analysis

The standard deviation of the APAIS score derived from the existing literature is 3.2^44^. Assuming that the APAIS value of the gum group compared to the control group must decrease by 2 points to detect clinically meaningful differences, the necessary sample size was 42 participants for each group with a power of 0.8 and an α value of 0.05. We decided that 47 patients in each group were to be enrolled to account for an expected 10% attrition rate, with a total of 94.

All data were tested for normality by the Shapiro–Wilk test and were presented as means (standard deviations [SD]) or as medians (interquartile ranges [IQR]). Differences between groups were analyzed using the chi-square test or Fisher's exact test for categorical variables, and Student’s t test or Wilcoxon’s rank sum test for continuous variables as appropriate. Post hoc analyses were also performed. Statistical significance was defined by *p* values < 0.05. All analyses were performed using SAS version 9.4 (SAS Institute Inc, Cary, NC, USA).

### Conference presentation

Preliminary data for this study were presented as a poster presentation at the KoreAnesthesia 2020. Nov 05–07, 2020, Incheon, Korea.

## Supplementary Information

Supplementary Information.
