# Supplementary material for: Anxiolytic effects of chewing gum during preoperative fasting and patient-centered outcome in female patients undergoing elective gynecologic surgery: randomized controlled study
Source: Sci Rep. 2022 Mar 9;12:4165. doi: 10.1038/s41598-022-07942-6 (PMC8907183; doi:10.1038/s41598-022-07942-6)

Supplemental Fig 1. Self - assessment NRS (Numeric rating scale) form; 0 =calm/ no anxiety to 10 extreme anxiety.

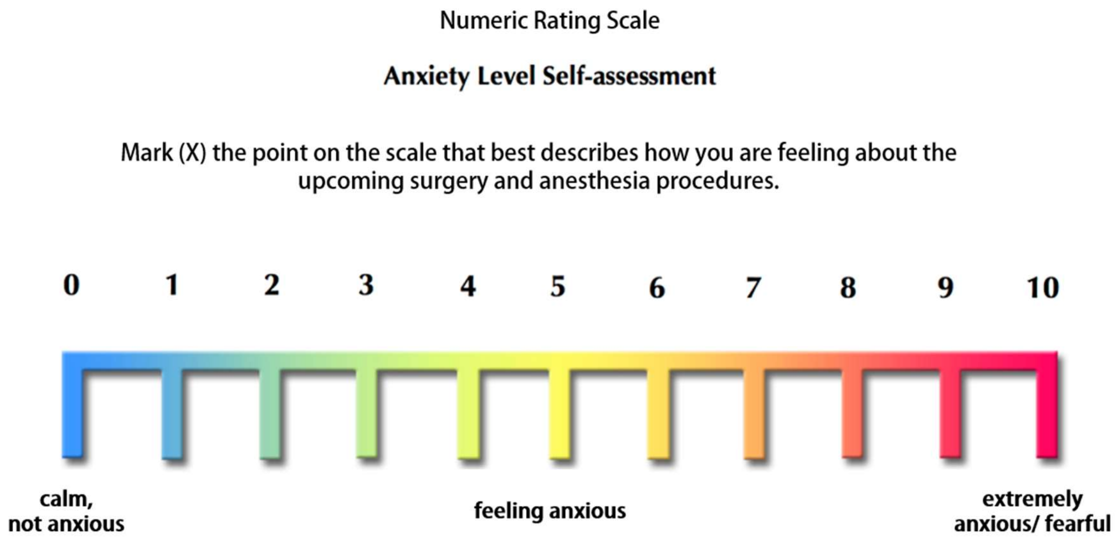

Supplement: Supplementary file 1 — Supplementary Information. [file 41598_2022_7942_MOESM1_ESM.pdf]
